# Using angiogenic factors and their soluble receptors to predict organ dysfunction in patients with disseminated intravascular coagulation associated with severe trauma

**DOI:** 10.1186/cc11032

**Published:** 2012-03-20

**Authors:** T Wada, S Jesmin, S Gando, S Zaedi, H Yokota

**Affiliations:** 1 Nippon Medical School, Tokyo, Japan; 2 National Center for Global Health and Medicine, Tokyo, Japan; 3 Hokkaido University Graduate School of Medicine, Sapporo, Japan

## Introduction

Disseminated intravascular coagulation (DIC) is observed after not only sepsis but also trauma. DIC is associated with concomitant activation of coagulofibrinolytic disorder and systemic inflammation with endothelial dysfunction and microvascular permeability. The angiogenic factors, including vascular endothelial growth factor (VEGF), angiopoietin (Ang), and their receptors, play crucial roles in angiogenesis and microvascular permeability. The aim of the study was to assess: the relationship between angiogenic factors, their soluble receptors and organ dysfunction associated with DIC precipitated by severe trauma; and the effects of DIC-induced platelet consumption, thrombin generation and tissue hypoxia on the expression of these factors and receptors.

## Methods

Fifty-seven patients with severe trauma were divided into two subgroups: 30 DIC patients and 27 non-DIC patients. The serum levels of angiogenic factors were measured on admission (day 1), day 3, and day 5. We compared serum levels of these angiogenic factors between with and without DIC groups and evaluated their predictive value for organ dysfunction and outcome.

## Results

DIC patients showed higher Sequential Organ Failure Assessment (SOFA) scores, soluble fibrin and lactate levels. The serum levels of VEGF, Ang1, and the sTie2 levels were lower in the DIC patients than the non-DIC patients. The serum levels of sVEGFR1, Ang2 and the Ang2/Ang1 ratio in the DIC patients were higher than in those without DIC. The sVEGFR2 levels showed no statistically significant difference between the patients with and without DIC. The levels of sVEGFR1, Ang2 and the Ang2/Ang1 ratio correlated with the SOFA score. In particular, sVEGFR1 and Ang2 were independent predictors of an increase in the SOFA score. The lactate levels independently predicted increases in the levels of sVEGFR1 and Ang2 and platelet consumption also independently predicted the increase in Ang2 levels in severe trauma patients with DIC.

## Conclusion

Angiogenic factors and their soluble receptors, particularly sVEGFR1, play pivotal roles in the development of organ dysfunction in DIC associated with severe trauma. The DIC-induced tissue hypoxia and platelet consumption plays crucial roles in inducing sVEGFR1 and Ang2, and in determining the prognosis of the severity of organ dysfunction.

